# Dissection of the *Candida albicans* Cdc4 protein reveals the involvement of domains in morphogenesis and cell flocculation

**DOI:** 10.1186/1423-0127-20-97

**Published:** 2013-12-20

**Authors:** Chuen Chin, Wei-Chung Lai, Tai-Lin Lee, Tzu-Ling Tseng, Jia-Ching Shieh

**Affiliations:** 1 Division of Infectious Disease, Department of Internal Medicine, Antai Medical Care Cooperation Antai Tian-Sheng Memorial Hospital, Pingtung, Taiwan; 2 Department of Biomedical Sciences, Chung Shan Medical University, No. 110, Sec. 1, Jianguo N. Road, Taichung City 40201, Taiwan; 3 Department of Molecular Biotechnology, Da-Yeh University, Changhua County, Taiwan; 4 Department of Medical Research, Chung Shan Medical University Hospital, Taichung City, Taiwan

**Keywords:** *Candida albicans*, *CDC4* domains, Morphogenesis, Flocculation

## Abstract

**Background:**

*CDC4*, which encodes an F-box protein that is a member of the Skp1-Cdc53/Cul1-F-box (SCF) ubiquitin E3 ligase, was initially identified in the budding yeast *Saccharomyces cerevisiae* as an essential gene for progression through G1-S transition of the cell cycle. Although *Candida albicans CDC4* (*CaCDC4*) can release the mitotic defect caused by the loss of *CDC4* in *S. cerevisiae*, *CaCDC4* is nonessential and suppresses filamentation.

**Results:**

To further elucidate the function of *CaCDC4*, a *C. albicans* strain, with one *CaCDC4* allele deleted and the other under the repressible *C. albicans MET3* promoter (*CaMET3*p) control, was made before introducing cassettes capable of doxycycline (Dox)-induced expression of various *C. albicans* Cdc4 (*Ca*Cdc4) domains. Cells from each strain could express a specific *Ca*Cdc4 domain under Dox-induced, but *CaMET3-CaCDC4* repressed conditions. Cells expressing domains without either the F-box or WD40-repeat exhibited filamentation and flocculation similarly to those lacking *CaCDC4* expression, indicating the functional essentiality of the F-box and WD40-repeat. Notably, cells expressing the N-terminal 85-amino acid truncated *Ca*Cdc4 partially reverse the filament-to-yeast and weaken the ability to flocculate compared to those expressing the full-length *Ca*Cdc4, suggesting that N-terminal 85-amino acid of *Ca*Cdc4 regulates both morphogenesis and flocculation.

**Conclusions:**

The F-box and the WD40-repeat of *Ca*Cdc4 are essential in inhibiting yeast-to-filament transition and flocculation. The N-terminal region (1–85) of *Ca*Cdc4 also has a positive role for its function, lost of which impairs both the ability to flocculate and to reverse filamentous growth in *C. albicans*.

## Background

*Candida albicans* is a natural diploid without a complete sexual cycle and exists as yeast, pseudohyphal, and hyphal cells [1]. It is capable of a morphological switch induced by environmental stimuli [2], essentially via cAMP-mediated and MAPK signaling pathways [3]. Importantly, its ability to alter morphology among cell types is associated with virulence to humans [4]. Many cell cycle regulators including cyclins are also known to control morphogenesis in *C. albicans*[5].

Recently, an F-box protein encoded *C. albicans CDC4* (*CaCDC4*) has been shown to play a role in filamentous development [6,7]. Cdc4, originally identified in the budding yeast *Saccharomyces cerevisiae*, encodes ubiquitin E3 ligases, which belongs to a member of the Skp1-Cdc53/Cul1-F-box (SCF) complex. This complex is known to play a role in ubiquitin-proteasome dependent degradation of regulatory proteins in eukaryotes [8]. A specific SCF complex is designated by its associated F-box protein. This protein is variable with two interacting domains of F-box for Skp1 and WD40-repeat (or LRR) for specific substrates [9], such that Cdc4 can be named SCF^Cdc4^. To progress through the G1-S transition in *S. cerevisiae*, SCF^Cdc4^ is required to degrade Sic1 [10] and Far1 [11], which are the cyclin-dependent kinase inhibitors. Therefore, *S. cerevisiae CDC4* (*ScCDC4*) is essential in *S. cerevisiae*.

Although *Ca*Cdc4 is a structural homolog of *S. cerevisiae* Cdc4 (*Sc*Cdc4) and is capable of rescuing the mitotic defect caused by the loss of *ScCDC4* in *S. cerevisiae*[7], the functions of *Ca*Cdc4 and ScCdc4 are dissimilar as the null *Cacdc4* mutant is viable and the depletion of *Ca*Cdc4 causes the accumulation of Sol1 (Sic1 like) for hyphal development rather than initiation of cell cycle arrest [6]. This verifies that *CaCDC4* is nonessential and suppresses filamentation and suggests that controlling the degradation on Sol1 in *C. albicans* by *Ca*Cdc4 is important for inhibition of filamentation. Therefore, while *C. albicans* Sol1 is likely a substrate of SCF^CaCdc4^, which can be demonstrated by the reduction of Sol1 when *Ca*Cdc4 is overexpressed [6], there has not been any direct evidence to support this hypothesis. Additionally, the filamentous properties for mutants of *Cacdc4* null and *Cacdc4 sol1* double null were comparable. This refutes the idea that Sol1 is the sole target of *Ca*Cdc4. Indeed, with an affinity-purification approach, we have isolated at least two novel *Ca*Cdc4-associated proteins [12] that are potential substrates of *Ca*Cdc4.

To further elucidate the role of *CaCDC4* and its mediation through a characteristic F-box protein of SCF ubiquitin E3 ligase in *C. albicans*, we have sought to dissect the *Ca*Cdc4 domains associated with filamentation. In this study, we made a *C. albicans* strain with one deleted *CaCDC4* allele and repressed the other by *CaMET3* promoter (*CaMET3*p) using methionine and cysteine (Met/Cys). We used this strain to introduce plasmids capable of inducing expression of various *Ca*Cdc4 domains with doxycycline (Dox). We observed the roles of F-box and WD40-repeat for *Ca*Cdc4 function and the possible role of the N-terminal 85-amino acid for morphogenesis. We also showed that *C. albicans* cells that lacked *Ca*Cdc4 triggered flocculation. Moreover, we found that N-terminal 85-amino acid of *Ca*Cdc4 is required for inhibition of both filamentation and flocculation.

## Methods

### Strains and growth conditions

*E. coli* strain DH5α was used for the routine manipulation of the plasmids. They were grown at 37°C in LB broth medium [13] or on plates containing 1.5% agar (Difco, BD Biosciences), with 50 μg/ml ampicillin or 30 μg/ml kanamycin. All *C. albicans* strains (Table 1) were derived from auxotrophic strain BWP17 (*arg4/arg4 his1/his1 ura3/ura3*) [14]. They were grown at 30°C in either yeast extract-peptone-dextrose (YEPD) or supplemented minimal synthetic defined (SD) medium with 2% glucose with or without 2% agar [15]. While Ura^+^ prototrophs were selected on SD agar plates without uridine, His^+^ prototrophs were selected on SD plates without histidine. Selection for the loss of the *C. albicans URA3* (*CaURA3*) marker was performed on plates with 50 μg/ml uridine and 1 mg/ml 5-fluoroorotic acid (5-FOA, MD Bio). To repress the *CaCDC4* expression that was controlled by *CaMET3*p, strains were grown on SD medium or on plates with 2.5 mM Met/Cys, which has been shown to optimally switch off the expression of the *CaMET3*p-driven downstream gene [16]. To induce gene expression under the Tet-on system, 40 μg/ml Dox (Sigma) was added to YEPD or SD media.

**Table 1:** ***Candida albicans *** **strains used in this study**

**Systemic name of the strain**	**Parental strain**	**Name relevant to genotype**	**Genotype**
BWP17		*CaCDC4* +/+	*ura3::imm434/ura3:imm434 his1::hisG / his1::hisG arg4::hisG/arg4::hisG*
JSCA0018	BWP17	*CaCDC4* +/*U*3-	*CaCDC4/cdc4::CaURA3-dpl200*
JSCA0021	JSCA0018	*CaCDC4 M*3/*U*3-	*Cacdc4::URA3-dpl200/P*_*MET3*_*-CaCDC4:HIS1*
JSCA0022	JSCA0021	*CaCDC4 M*3/-	*Cacdc4::dpl200/P*_*MET3*_*-CaCDC4:HIS1*
JSCA0023	JSCA0022	*CaCDC4 M*3/- ∣ Tet-*CaCDC4*	*Cacdc4::dpl200/P*_*MET3*_*-CaCDC4:HIS1* ∣ *CaADH1/adh1::P*_*TET*_*-CaCDC4*:*CaURA3*
JSCA0024	JSCA0022	*CaCDC4 M*3/- ∣ Tet-*CaCDC4*-6HF	*Cacdc4::dpl200/P*_*MET3*_*-CaCDC4:HIS1* ∣ *CaADH1/Caadh1::P*_*TET*_*-CaCDC4-6HF*:*CaURA3*
JSCA0025	JSCA0022	*CaCDC4 M*3/- ∣ Tet-ΔN-6HF	*Cacdc4::dpl200/P*_*MET3*_*-CaCDC4:HIS1* ∣ *CaADH1/Caadh1::P*_*TET*_*-CaCDC4(85–768)-6HF*:*CaURA3*
JSCA0026	JSCA0022	*CaCDC4 M*3/- ∣ Tet-F-box-6HF	*Cacdc4::dpl200/P*_*MET3*_*-CaCDC4:HIS1* ∣ *CaADH1/Caadh1::P*_*TET*_*-CaCDC4(241–392)-6HF*:*CaURA3*
JSCA0027	JSCA0022	*CaCDC4 M3*/- ∣ Tet-WD40-6HF	*Cacdc4::dpl200/P*_*MET3*_*-CaCDC4:HIS1* ∣ *CaADH1/adh1::P*_*TET*_*-CaCDC4(393–768)-6HF*:*CaURA3*
JSCA0030	JSCA0022	*CaCDC4 M3*/- ∣ Tet-ΔNF-6HF	*Cacdc4::dpl200/P*_*MET3*_*-CaCDC4:HIS1* ∣*CaADH1/Caadh1::P*_*TET*_*-CaCDC4(85–392)-6HF*:*CaURA3*

### Plasmid DNA manipulation

Plasmid DNA was extracted routinely from *E. coli* cultures using Gene-Spin^TM^ MiniPrep purification Kit-V^2^ (PRO TECH, Taipei, Taiwan) and the instructions provided by the manufacturer. *E. coli* was transformed with plasmid DNA by using CaCl_2_. The DNA cassettes were introduced into *C. albicans* by the lithium acetate method as described previously [17].

### Construction of *C. albicans* strains

**Figure 1:**
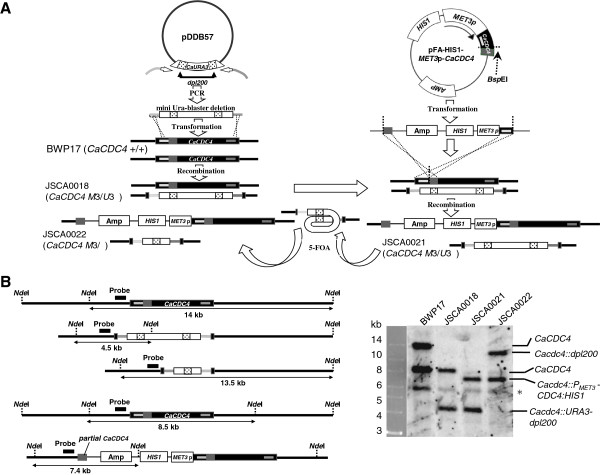
**Construction of a *C. albicans *strain for repressibly expressing *CaCDC4*. (A)** Strain construction (detailed in the Methods). The first *CaCDC4* allele on BWP17 was deleted by mini-Ura-blaster to obtain JSCA0018. Plasmid pFA-HIS1-MET3p-CaCDC4 containing partial *CaCDC4* coding sequence was linearized at a unique site for introducing into strain JSCA0018 to generate JSCA0021. 5-FOA was used to counter-select *CaURA3* removal to obtain JSCA0022 for re-introducing the Tet-on plasmid with a *CaURA3* marker. **(B)** Verification of constructed strains by Southern blotting analysis. Organization of the *CaCDC4* locus with respect to *Nde*I sites is shown. The relative positions of the probe used and the predicted *Nde*I-digested pattern of the *CaCDC4* locus are indicated. Two *Nde*I-fragments of 14 kb and 8.5 kb, specific to *CaCDC4*, could be detected in genomic DNA from BWP17 digested with *Nde*I; two *Nde*I-fragments of 8.5 kb and 4.5 kb, specific to *CaCDC4* and *Cacdc4::URA3-dpl200*, respectively, could be detected in JCSA0018; two *Nde*I-fragments of 4.5 kb and 7.4 kb specific to *Cacdc4::URA3-dpl200* and *Cacdc4::P*_*MET3*_*-CDC4:HIS1*, respectively, could be detected in JSCA0021; and two *Nde*I-fragments of 13.5 kb and 7.4 kb specific to *Cacdc4::dpl200* and *Cacdc4::P*_*MET3*_*-CDC4:HIS1*, respectively, could be detected in JSCA0022. A non-specific *Nde*I-fragment is indicated as “*” can be detected in all strains tested.

Initially, a strain with repressed *CaCDC4* expression was made. A mini-Ura-blaster cassette, flanked with 60-bp sequences homologous to *CaCDC4,* was PCR-amplified using a template of plasmid pDDB57 and long primers of CaCDC4-URA3-F and CaCDC4-URA3-R (Table 1). BWP17 was transformed by integration of the cassette into the *CaCDC4* locus to generate Ura^+^ strain JSCA0018. The plasmid pFA-HIS1-MET3p-CaCDC4, with a partial *CaCDC4* coding sequence for N-terminal *Ca*Cdc4 (1–563), was linearized with *Bsp*EI and used to transform JSCA0018 to generate His^+^ JSCA0021 (Figure 1A; Table 1). Cells of JSCA0021 were plated with 5-FOA to induce recombination between two copies of *dpl200* flanking the mini-Ura-blaster for a loss of *CaURA3* to generate JSCA0022.

To allow the expression of cassettes encoding assorted *Ca*Cdc4 domains in *C. albicans*, a Tet-on plasmid, pTET25M [18], which is derived from pTET25 [19] for inducing gene expression with Dox, has been developed. To regulate *CaCDC4* expression by the Tet-on system, the coding sequence of *CaCDC4* was PCR-amplified using plasmid CaCDC4-SBTA bearing *CaCDC4* (Lai WC, unpublished results), primers CaCDC4-SalI and CaCDC4-BglII (Table 2), and *Pfu* polymerase (5 U/μl, MD bio), digested with *Sal*I and *Bgl*II for cloning into pTET25M, from which pTET25M-CaCDC4 was generated. Moreover, *CaCDC4*-6HF, which encodes 6×histidine and FLAG (6HF) tags at the C-terminal of *Ca*Cdc4, was PCR-amplified with primers CaCDC4-6HF SalI and CaCDC4-6HF BglII (Table 2), followed by digestion with *Sal*I and *Bgl*II and cloning into pTET25M to obtain pTET25M-CaCDC4-6HF.

**Table 2:** Oligonucleotides used in this study

**Name**	**Sequence**^**a**^
CaCDC4 XhoI F	GAA*CTCGAGA*TGGATAAGAAATCAAAG
CaCDC4 XhoI R	GAA*CTCGAG*CTGTAAAAGTGGTTGACT
CaCDC4 SalI	TAGC*GTCGAC*ATGGATAAGAAATCAAAGC
CaCDC4 BglII	TCG*AGATCT*TCACTGTAAAAGTGGTTGAC
CaURA3-dpl200 BamHI	AAT*GGATCC*CCAGATATTGAAGGTAAAAGG
CaURA3-dpl200 XhoI	ATT*CTCGAG*CTAGAAGGACCACCTTTGAT
TET25M KpnI	CAA*GGTACC*GAACCATCGTGAGTGTAA
TET25M BamHI	GAA*GGATCC*CGACATTTTATGATGGAA
CaCDC4-6HF SalI	GCGT*GTCGAC*GTCATGGATAAGAAATCAAAGCTA
CaCDC4-6HF^**b**^ BglII	TCG*AGATCT*ttatttatcatcatcatctttataatcACCACCgtggtggtggtggtggtg*CTCGAG CGGCCGC*TGTAAAAGTGGTTGACTGAAATC
CaCDC4 ΔN AatII	AATA*GACGTC*CTTATGCCCTCATGTGACGAC
CaCDC4 ΔN XhoI	ATC*CTCGAG*CTGTAAAAGTGGTTGACTGA
CaCDC4 F-box AatII	AAGC*GACGTC*ATGAGCAATGAACCTACT
CaCDC4 F-box XhoI	GCCA*CTCGAG*CCACCTATTGACAATTAT
CaCDC4 WD40 AatII	GCTA*GACGTC*ATGGATCCAAAGTTCAAAC
CaCDC4-URA3-F	ATGGATAAGAAATCAAAGCTATTCAAATATCCTTTGAGCGAGGAGACGGCTAAATTTGAGGTTTTCCCAGTCACGACGTT
CaCDC4-URA3-R	TCACTGTAAAAGTGGTTGACTGAAATCTAGAATCTCAATAAACGTTTCACCTTCATCTTCTGTGGAATTGTGAGCGGATA
CaADH1_probe_F	GGAGTATTGGCATTGTTGGG
CaADH1_probe_R	AAGCTTGCTTGCATGACGAG
CaCDC4_probe_F	GGTTTCCAACACTTTCCCAG
CaCDC4_probe_R	CACTACTAGTTGGTTGCTGT

^**a**^Restriction enzyme sites are in italics.

^**b**^Sequences complementary to those encoding 6×His and FLAG are in lower case letters. The italics has been used for restriction enzymes as in note “a”. The underline is new replaced with lower case letters.

**Figure 2:**
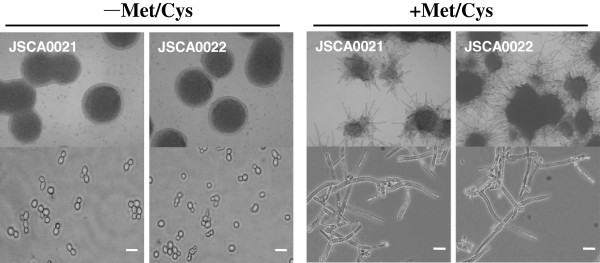
**Morphological analysis of the constructed *CaCDC4 *repressible strains.** Cells of strains JSCA0021 and JSCA0022 were grown on SD medium or plates with (+) or without (-) Met/Cys. Colonies were photographed with MEIJI stereoscopic microscope EMZ5 at 40× magnification (top panel). Cells in liquid culture were visualized and recorded with a Nikon 50i microscope at 400× magnification (bottom panel). Bars represent 10 μm.

To define the function of the distinct *Ca*Cdc4 domains (Figure 2A), different *CaCDC4* portions were used to replace the full length *CaCDC4* coding sequence on pTET25M-CaCDC4-6HF. By using the primer sets listed in Table 2, the following constructs were made: pTET25M-ΔNCaCDC4-6HF (with primers CaCDC4 ΔN AatII and CaCDC4 ΔN XhoI), which encodes the N-terminal truncated *Ca*Cdc4; pTET25M-F-6HF (with primers CaCDC4 F-box AatII and CaCDC4 F-box XhoI), which encodes the F-box domain with flanking regions; pTET25M-WD40-6HF (with primers CaCDC4 WD40 AatII and CaCDC4 ΔN XhoI), which encodes eight copies of WD40-repeat; and pTET25M-ΔNF-6HF (with primers CaCDC4 ΔN AatII and CaCDC4 F-box XhoI), which encodes truncated N-terminal *Ca*Cdc4 and the F-box domain. All inserts of the constructs were released with *Aat*II and *Xho*I to replace the full-length *CaCDC4* on pTET25M-CaCDC4-6HF. Consequently, plasmids bearing those *CaCDC4* segments flanked with common *C. albicans ADH1* (*CaADH1*) sites were digested with *Sac*II and *Kpn*I, each of which was transformed into *C. albicans* for integration at the *CaADH1* locus. All strains were verified by colony PCR with specific primers before subjecting to Southern blotting analysis.

### Southern blotting analysis

Genomic DNA from the *C. albicans* strains was isolated by the MasterPure™ Yeast DNA Purification Kit (Epicentre®, an Illumina company) according to the manufacture’s instruction. Southern blotting was performed with the aid of the Rapid Downward Transfer System (TurboBlotter™, Whatman) using 10 μg of the restriction enzyme-digested genomic DNA. The DNA on the blot was hybridized with a probe amplified by the PCR DIG probe synthesis kit (Roche) with the primers CaCDC4_Probe_F and CaCDC4_Probe_R for *CaCDC4* locus or CaADH1 Probe_F and CaADH1 probe_R for *ADH1* locus (Table 2) using DIG Easy Hyb (Roche). To reveal the structure of gene locus, the DIG Luminescent Detection Kit (Roche) was used after hybridization, and the luminescent images of blot were captured with the imaging analysis system (ImageQuant LAS4000 mini, GE Healthcare Life Sciences).

### Protein extraction and Western blot analysis

Cultured cells were collected, and the total protein from each sample was extracted as described previously [20]. The proteins were resolved by 10% SDS-PAGE and transferred to PVDF membranes (PerkinElmer, Boston, USA). Proteins on the membranes were probed with polyclonal antibody to FLAG (Sigma) in 1:2000 dilution and detected using the SuperSignal West Pico Chemiluminescent Substrate Kit (PIERCE). These were recorded with the Luminescent Image Analyzer (FUJIFILM LAS-1000) and analyzed by ImageGauge 3.46 and L Process v 1.96 (FUJIFILM).

### Flocculation assay by low-speed centrifugation

The cells of strains were streaked on YPD agar plate for 3 days and colonies were picked and inoculated into SD medium with required supplements for 48 hrs. Next, the cultures were diluted into fresh SD medium to 0.1 of an initial OD_600_ with required supplements. To simultaneously repress the expression of *CaMET3*p-driven *CaCDC4* and to induce the expression of various *CaCDC4* segments encoding series of *Ca*Cdc4 domains, 2.5 mM Met/Cys and 40 μg/ml Dox were also added into the SD medium. After 48 hrs, the cultures were spun down for 1 minute at 500 rpm, and the suspensions of the cultures were sampled to determine their optical density at OD_600_. Three independent assays were conducted and each sample was assayed in duplication. A paired Student t test with p < 0.05 was considered significance.

### Ca^2+^-initiated flocculation assay

The *FLO*-encoded flocculins are known to be essential for flocculation in *S. cerevisiae*[21]. Functional homologues of *FLO* genes have been found in *C. albicans*. In particular, the important *S. cerevisiae* gene *FLO11* responsible for flocculation has *C. albicans* functional counterpart *ALS1*[22]. Since *FLO11*-associated flocculation is dependent on the presence of Ca^2+^, we adopted an alternative flocculation assay in which the rate of flocculation is initiated by Ca^2+^ and the optical density was assessed within a short time-frame [23]. Briefly, to initiate flocculation, an aliquot of 800 μl deflocculated cell suspension was transferred into a 1-ml cuvette, followed by addition of 200 μl of 100 mM CaCl_2_. The cuvette was mixed robustly by pipetting and the absorbance (OD_600_) was assessed instantly at 30-s intervals for 5 minutes using a spectrophotometer (DU800, Beckman Coulter, Inc.). All assays were conducted in triplicate.

## Results

### Constructing a *C. albicans* strain capable of conditionally repressing the expression of *CaCDC4*

To establish *C. albicans* strains capable of expressing *CaCDC4* and its domains solely controlled under a *Tet* promoter directly in *C. albicans*, BWP17, with both alleles of *CaCDC4* deleted, was constructed to accommodate Tet-on plasmid cassettes capable of expressing assorted *Ca*Cdc4 domains induced by Dox. The first allele of *CaCDC4* was deleted in BWP17 by mini-Ura-blaster [24] to generate the JSCA0018 strain (Figure 1A; Table 1). This strain was used to delete the second *CaCDC4* allele to obtain a *Cacdc4* null mutant. However, *Cacdc4* null mutant cells growing as filamentous form with toughened cell walls obstructed transformation.

To overcome this problem, the strain JSCA0021 (Figure 1A; Table 1) was created that had one *CaCDC4* allele deleted and the other under *CaMET3* control that was Met/Cys repressible. To allow the introduction of Tet-on cassettes with the same *CaURA3* selectable marker as the mini-Ura-blaster on JSCA0021, 5-FOA was used as a counter-selection agent to remove *CaURA3* from JSCA0021, from which JSCA0022 was obtained (Figure 1A; Table 1). The strains were PCR-confirmed with specific primers before subjecting to Southern blotting analysis. The *CaCDC4* locus from BWP17 strain could detect two *Nde*I-digested fragments with size of 14 kb and 8.5 kb, respectively (Figure 1B). The size shifting of *Nde*I-fragment flanking *CaCDC4* from 14 kb to 4.5 kb demonstrated that one *CaCDC4* allele was integrated with the mini-Ura-blaster cassette as in strain JSCA0018 (Figure 1B). The size shifting of *Nde*I-fragment flanking *CaCDC4* from 8.5 kb to 7.4 kb demonstrated that the other *CaCDC4* allele integrated with the *MET3*-diven *CaCDC4* plasmid as in strain JSCA0021 (Figure 1B). Strain JSCA0021 could be further popped out the mini-Ura-blaster cassette to obtain strain JSCA0022 in which the size shifting of *Nde*I-fragment flanking *CaCDC4* from 4.5 kb to 13.5 kb (Figure 1B). These results indicate that all strains constructed have expected organizations in their genome.

### Phenotypic verification of *C. albicans* strains capable of conditionally repressing the expression of *CaCDC4*

It has been shown that Ura^—^ auxotrophic mutants are avirulent [25] and other virulence-associated features can be influenced by the level of *CaURA3* gene expression [26]. To assess presence of *CaURA3* having effect on yeast-to-filament transition, the yeast-to-filament transitions between strain JSCA0021 and JSCA0022 were compared, cells of those strains were assessed under *CaMET3*p repressed or de-repressed conditions. Cells of both strains on SD plates without Met/Cys grew as circular colonies with smooth surfaces (Figure 2). By contrast, cells on plates with Met/Cys formed irregular colonies with filaments (Figure 2). Under the microscope, these strains exhibited equivalent filamentous forms, suggesting a comparable ability to deplete *CaCDC4* for expression and inability of *CaURA3* interfering with yeast-to-filament transition *in C. albicans*. Subsequently, JSCA0022 was used as a parental strain to introduce the Tet-on cassettes (with *CaURA3* marker) that encoded assorted *Ca*Cdc4 domains.

### Establishment of Tet-on cassettes capable of expressing assorted *CaCDC4* domains in *C. albicans* reveals that both the F-box and WD40-repeat are required for *Ca*Cdc4 function

**Figure 3:**
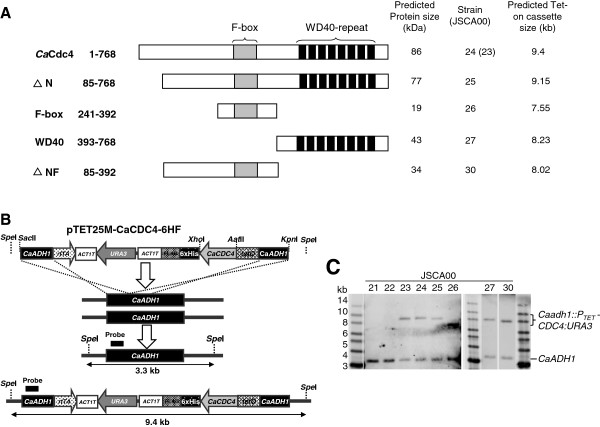
**Construction of *C. albicans *strains for Dox-inducing the expression of assorted *CaCDC4 *domains. (A)** Schematic representation of *Ca*Cdc4 domains expressed from the Tet-on system. The strains with which they are derived and predicted Tet-on cassette size are shown **(B)** Generation of Tet-on cassettes for expressing assorted CaCdc4 domains. Different portions of *CaCDC4* were PCR-generated with primer sets (Table 2) containing common *Aat*II and *Xho*I sites for replacing full-length *CaCDC4* on pTET25M-CaCDC4-6HF as described in the Methods. By digestion with *Sac*II and *Kpn*I, each cassette was used to transform *C. albicans* strain JSCA0022 for integration into the *CaADH1* locus. (**C**) Verification of Tet-on cassettes being integrated into *CaADH1* locus by Southern blotting analysis. Organization of the *CaADH1* locus with respect to *Spe*I sites is shown in Figure 3B. The relative positions of the probe used and the predicted *Spe*I-digested pattern of the *CaADHI* locus are indicated in Figure 3B. One *Spe*I-fragment of 3.3 kb specific to *CaADH1* locus could be detected in genomic DNA from strain JSCA0022 and its derivatives digested with *Spe*I. The correctness of integration of the cassette into the *CaADH1* locus of various strains was confirmed by alteration of the *Spe*I-fragment from size of 3.3 kb to 9.4 kb (Figure 3B) or various sizes as indicated in Figure 3A.

The filamentous development of JSCA0022 under *CaMET3*p-*CaCDC4* repressed conditions, with Met/Cys and the Tet-on system, allows us to study the function of the *Ca*Cdc4 domains. A set of Tet-on cassettes (obtained from pTET25M-CaCDC4-6HF, pTET25M-ΔN-6HF, pTET25M-F-box-6HF, pTET25M-WD40-6HF, and pTET25M-ΔNF-6HF) that encoded each of the assorted domains of *Ca*Cdc4 (Figure 3A) were used to transform JSCA0022 (which contained a *CaMET3*p-repressible *CaCDC4*) to Ura^+^ by integration at the *CaADH1* locus (Figure 3B). The correctness of the strains was confirmed by yeast colony PCR with specific primers before Southern blotting analysis. The *CaADH1* locus from strain JSCA0022 could detect a *Spe*I-digested fragment with size of 3.3 kb (Figure 3C). The *CaADH1* locus from strains JSCA0023 and JSCA0024 detected an increased *Spe*I-digested fragment of 9.4 kb due to the integration of Tet-on cassettes of either pTET25M-CaCDC4 or pTET25M-CaCDC4-6HF (Figure 3C). The *CaADH1* locus from other strains also showed expected alteration in size according to the size of different *CaCDC4* domains (Figure 3C). These results confirmed the correctness of the strains.

**Figure 4:**
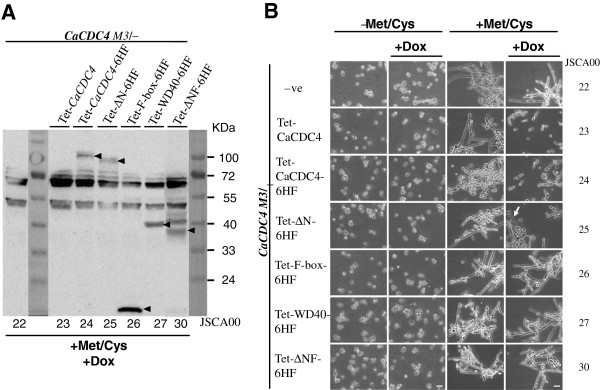
**Morphological analysis of *C. albicans *strains capable of Dox-inducing the expression of assorted *CaCDC4 *domains.** Cells were grown initially in SD medium without Met/Cys to saturation and were diluted to the same initial concentration. Cells were grown exponentially in SD in the absence of 2.5 mM Met/Cys, with or without 50 μg/ml Dox (-Met/Cys + Dox or -Met/Cys), or in the presence of 2.5 mM Met/Cys, with or without 50 μg/ml Dox (+Met/Cys + Dox or + Met/Cys). **(A)** The Dox-inducibly expressing assorted *Ca*Cdc4 protein domains under *CaMET3*-*CaCDC4* repressed conditions was verified by Western blotting with polyclonal antibody to FLAG. The non-specific signals between 72 and 55 kDa, and between 55 and 40 kDa are served as a loading controls. **(B)** The images were visualized and recorded with a Nikon 50i microscope at 400× magnification. The arrow in white indicates filamentous cells. Bars represent 10 μm. The designations of strains are the same as in Table 1.

The JSCA0022 strain, which expressed the non-tagged and repressible *Ca*Cdc4, was used as a negative control. The sample obtained from JSCA0022 contained two prominent proteins of approximately 55 kDa and 72 kDa (Figure 4A) which were presumably a result of cross-reactivity to the anti-FLAG antibody. Those two proteins were used as an internal control. The F-box and WD40-repeat proteins from strains JSCA0026 and JSCA0027 migrated to their expected positions of approximately 19 kDa and 43 kDa (Figure 4A), respectively. However, the full-length *Ca*Cdc4 and the N-terminus truncated *Ca*Cdc4 (ΔN) from strains JSCA0024 and JSCA0025 exhibited signals at positions corresponding to 100 kDa and over 100 kDa (Figure 4A), respectively, as opposed to 86 kDa and 77 kDa, respectively. Three distinctive signals (Figure 4A) were observed for strain JSCA0030 expressing ΔNF of *Ca*Cdc4, but none of them matched the expected size of 34 kDa; however, the signal at the lowest position could be meaningful. These patterns of expression were similar to strains expressing each of the domains, with either BWP17 or JSCA0021 as a parental strain (Lai WC, unpublished results). Therefore, even though some of the strains expressed domains with unexpected size, they were unique from the negative control of JSCA0022. We concluded that the Tet-on system functions in JSCA0022 and that *Ca*Cdc4 might be undergoing undefined modifications.

To determine the function of the assorted *Ca*Cdc4 domains, JSCA0022-based strains capable of repressing *CaCDC4* and inducing expression of assorted *Ca*Cdc4 domains were grown in SD medium with or without Met/Cys and in the presence or absence of Dox. Cells from strains in SD medium without Met/Cys grew as yeast in the presence or absence of Dox (Figure 4B). By contrast, cells from strains in medium with Met/Cys grew with filaments (Figure 4B). As expected, cells of JSCA0023 and JSCA0024 growing on medium with Met/Cys and Dox and that expressed the full-length *Ca*Cdc4 with or without tag grew as yeast. Disregarding the full-length *Ca*Cdc4, cells from all strains, except JSCA0025 expressing assorted domains, still grew as filaments (Figure 4B). Under Met/Cys and Dox conditions, cells from JSCA0025 expressing the N-terminal 85-amino acid truncated *Ca*Cdc4 seemed to have an ability to suppress filamentation but not complete back to the yeast form (Figure 4B). This is in consistent with our previous observation in which, comparing with cells capable of expressing the full-length *Ca*Cdc4 under the *CaMET3*p repressible control, those cells expressing the N-terminal 85-amino acid truncated *Ca*Cdc4 lagged behind in reaching exponential stage (Additional file 1: Figure S1) and converted to filamentous form earlier (Additional file 2: Figure S2) in the repressed condition.

### *C. albicans CDC4* negatively regulating cell flocculation

**Figure 5:**
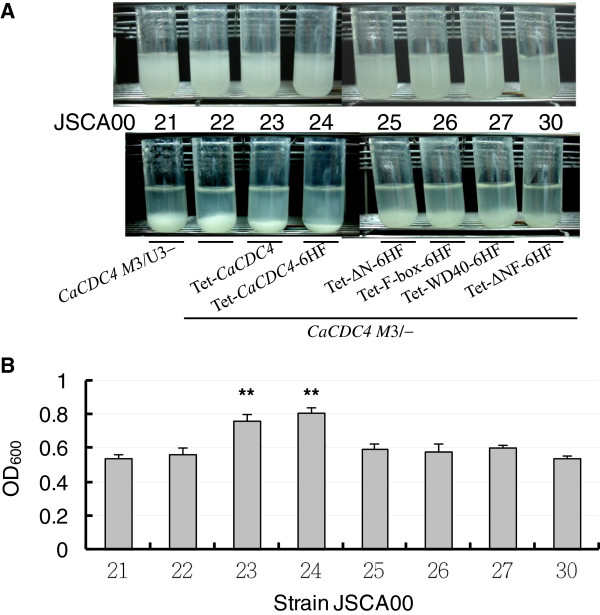
**Analysis of cell flocculation by low-speed centrifugation.** Cultures of the indicated strains were grown in SD medium with histidine, arginine, uridine for 2 days before diluting into the SD medium to an initial OD_600_ = 0.1 with addition of 2.5 mM Met/Cys to repress the expression of *CaMET3*p-driven *CaCDC4* and 40 μg/ml Dox to induce the expression of *Ca*Cdc4 domains tested for 48 hrs to OD_600_ ≈ 1.6. Cultures were photographed before and after centrifugation. **(A)**. A representative of the cultures. Upper panel: two-day culture. Bottom panel: cultures being spun down with 500 rpm for 1 minute. **(B)**. Quantitative results. Data are represented as means with standard deviation from three independent experiments, each sample was in duplication. The data from JSCA0022 were compared with those of other strains. **: P < 0.01. The designations of strains are the same as in Table 1.

Significant differences in the ability among strains to form suspensions (to resist flocculation) were observed. The extent of flocculation among strains was observed after resuspending the cells in cuvettes, where they remained for 30 seconds. When cells were grown under the Met/Cys and Dox conditions, only those from JSCA0023 and JSCA0024 were somewhat easier to maintain as a suspension. To exclude the possibility that this was a result of increases in cell density, cells from all strains were initially grown to saturation, and the cultures were subsequently diluted to the same initial optical density and grown exponentially to similar optical density. The extent of flocculation among strains was observed after spinning the cells for 1 minute at 500 rpm. The suspended cells were sampled for determination of their optical density. Cells resisted in flocculation would remain in suspension upon centrifugation. Under the *CaMET3*p de-repressed condition and in the presence or absence of Dox, all strains exhibited a similar degree of suspension. However, under the *CaMET3*p repressed condition, JSCA0026, JSCA0027, and JSCA0030 displayed flocculation similar to JSCA0022 regardless of the presence or absence of Dox (Figure 5A). While more cells of strains JSCA0023, JSCA0024 maintained as suspension, those of JSCA0025 with some filamentous cells, showed comparable extent of flocculation to JSCA0022 under *CaMET3*p repressed but Tet-on induced conditions (Figure 5).

**Figure 6:**
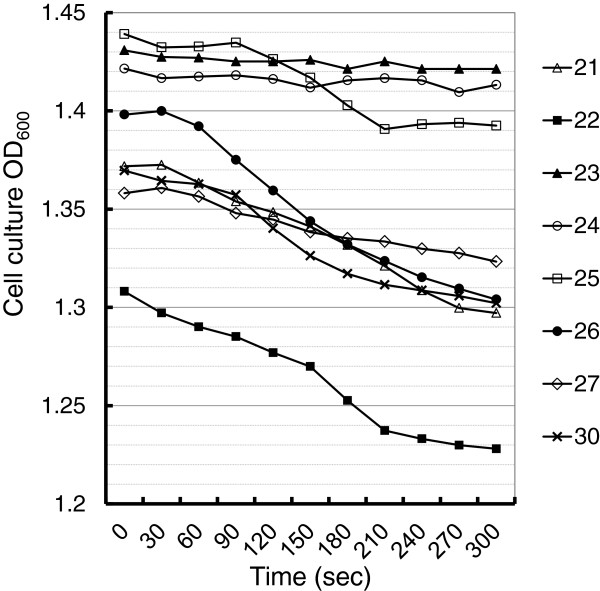
**Analysis of Ca^2+^ initiated cell flocculation.** The strains were grown as described in Figure 5 to saturation at OD_600_ ≈ 1.4. The cultures were harvested and washed twice with deflocculation buffer, followed by initiation of flocculation as described in the Methods. The assays were conducted in triplicate. The name of each strain shows only the last two numbers.

To solidify our observations, an alternative flocculation assay where flocculation is initiated by addition of Ca^2+^ to the culture medium being depleted with Ca^2+^ beforehand was used [23]. Only cells of JSCA0023 and JSCA0024 remained resistance in flocculation during the time-frame of 5-minute assay compared with those of the rest of strains (Figure 6), which were consistent with the results shown in Figure 5. However, both strains JSCA0025 and JSCA0027 exhibited greater ability to resist flocculation than that of JCSA 0026 and JSCA0030 when considering the differences in OD_600_ from the initial to the end points.

## Discussion

In this study, we aimed to dissect the function of *Ca*Cdc4 domains by introducing a Tet-on system with cassettes that encoded for a variety of *Ca*Cdc4 domains in a *C. albicans* mutant of *Cacdc4* null. However, the *Cacdc4* null mutant with a filamentous form could not be easily used to introduce the Tet-on cassettes; therefore, we constructed the JSCA0022 strain, where *CaURA3* was released from the strain JSCA0021, and *CaCDC4* expression was repressible. Under repressed conditions, the JSCA0022 strain showed similar filamentous morphology (Figure 2) to those from previous reports of cells with *CaCDC4* repressed strain [6,7] and of *cacdc4* null mutant [6] (Tseng TL, Hsu WH, and Shieh JC, unpublished results). We confirmed that the JSCA0022 strain under repressed conditions was equivalent to a strain that had completely lost *CaCDC4* function. Hence, by introduction of the Tet-on cassettes into JCSA0022 strain, each of the strains was capable of expressing individual *Ca*Cdc4 domains in the presence of Met/Cys and Dox for functional comparisons.

To verify the ability of the Tet-on cassettes in *C. albicans*, each of the cassettes encoding various *Ca*Cdc4 domains was transformed into BWP17 and JSCA0021 before introducing them into JSCA0022 at the *CaADH1* locus. Individual *Ca*Cdc4 domains from relevant strains were all detectable, suggesting that the Tet-on system functions in *C. albicans*. However, while cells expressing the F-box and the WD40 repeat could be detected as their expected sizes, those expressing the full-length *Ca*Cdc4, the N-terminus truncated *Ca*Cdc4 (ΔN), and the ΔNF of *Ca*Cdc4 could be detected at positions higher than anticipated (Figure 4A). In particular, the sample from strain JSCA0030 expressing the ΔNF could be detected three signals (Figure 4A), all of which were greater than the predicted sizes. These results suggest that the N-terminal *Ca*Cdc4 from residue 85 to 241 (Figure 3A) might be undergoing post-translational modification during the Tet-on-induced expression, although its functional significance is unknown. Interestingly, the region between residue 85 and 241 of *Ca*Cdc4 contains abundant serine and threonine residues, the majority of which are homologous to *S. cerevisiae* Cdc4 [7]. This implies possible phosphorylations or other modifications on these residues that is specific to *C. albicans.* However, the genuine nature of these residues remains to be determined, and their functional significance of this N-terminal *Ca*Cdc4 requires further study.

With regards to integration of *CaADH1* locus by the Tet-on cassette, it is known that *C. albicans adh1* homozygous null mutant gains the ability to form biofilm both *in vitro* and *in vivo*[27], suggesting a possible role of *CaADH1* in flocculation. However, the heterozygous *CaADH1* null mutant with which the homozygous *adh1* null mutant is reintegrated a functional copy of *CaADH1* to the *CaADH1* locus appears to be similar in biofilm formation as its isogenic wild-type strain. In addition, disruption of *CaADH1* has no consequence of morphology alteration in *C. albicans*[27] (Lai WC, unpublished results). Therefore, the possible effect of Tet-on cassette on flocculation and filamentation by integration, hence disruption of a copy of *CaADH1* locus can be excluded.

Under the Met/Cys and Dox conditions, cells expressing F-box, WD40 repeat, and the ΔNF of *Ca*Cdc4 exhibited filamentous forms similar to those of JSCA0022, whose *CaCDC4* was repressed, compared to those expressing the full-length *Ca*Cdc4 without or with tag (JSCA0023 and JSCA0024), which exhibited yeast forms (Figure 4B). These results suggest that both the WD40 repeat and F-box are essential to suppress the yeast-to-filament transition. Cells from strain JSCA0025 expressing the ΔN of *Ca*Cdc4, which were grown in the presence of Met/Cys and Dox, were only partially able to reverse filamentous cells to yeast cells, suggesting that the N-terminal 85-amino acid of *Ca*Cdc4 plays a role in the yeast-to-filament transition in *C. albicans*. The role of the N-terminal 85-amino acid of *Ca*Cdc4 for growth was observed previously, in which cells expressing N-terminal 85-amino acid truncated *Ca*Cdc4 lagged slightly in proliferation during the exponential stage (Additional file 1: Figure S1), and repression of the expression of the N-terminal 85-amino acid truncated *Ca*Cdc4 resulted in prominently lagging behind in growth, which was presumably due to the morphological alteration of cells to filaments in advance (Additional file 2: Figure S2) that delays proliferation as compared to those of yeast cells. Since the N-terminal 85-amino acid of *Ca*Cdc4 is unique compared to that of the *S. cerevisiae* Cdc4 [7], our finding reveals a role of N-terminal 85-amino acid of *Ca*Cdc4 on morphogenesis, which is unknown previously.

Importantly, cells of all JSCA0022-based strains exhibited flocculation in medium with Met/Cys, but the strains JSCA0023 (CaCDC4) and JSCA0024 (CaCDC4-6HF) exhibited less flocculation by adding Dox simultaneously (Figure 5). Unlike cells of JSCA0023 and JSCA0024, those of JSCA0025 expressing N-terminal 85-amino acid truncated *Ca*Cdc4 were unable to totally overturn filamentous-to-yeast cells, suggesting that N-terminal 85-amino acid is required for full activity of *CaCDC4* function in *C. albicans* to inhibit filamentation*.* However, if flocculation is tightly associated with filamentation, we expect to see the extent of flocculation in JCSA0025 (ΔN 6HF) being greater than that of JSCA0022 but less than that of JSCA0023 and JSCA0024 in the presence of Met/Cys and Dox. This was not revealed by the low speed-centrifugation method but by the Ca^2+^-initiation assay. Importantly, both JSCA0025 and JSCA0027 expressing *Ca*Cdc4 lacking N-terminal 85-amino acid (Figure 3A) exhibits similar extent of flocculation. Moreover, JSCA0025 that expressing *Ca*Cdc4 lacking N-terminal 85-amino acid could only partially suppress filamentation yet JSCA0027 that expressing *Ca*Cdc4 lacking N-terminal 85-amino acid and F-box with flanking regions completely lose the ability to inhibit filamentation (Figure 3A and Figure 4B). These results imply that N-terminal 85-amino acid of *Ca*Cdc4 has a role in inhibition of cell flocculation in *C. albicans* and that the F-box and its flanking region in addition to the N-terminal 85-amino acid of *Ca*Cdc4 might be associated with proper control of both morphogenesis and flocculation.

## Conclusions

Therefore, we conclude that F-box and WD40-repeat are important in suppressing yeast-to-filament transition and flocculation and that the N-terminal region (1–85) has a positive role in *CaCDC4* function, lost of which impairs reverse of filament-to-yeast and reduces the ability to flocculate in *C. albicans*. Moreover, the function of *Ca*Cdc4 for suppressing flocculation that is related to cell-cell adhesion [21] implies a role of *CaCDC4* in biofilm formation [28] that is under investigation.

## Abbreviations

SCF: Skp1-Cdc53/Cul1-F-box; CaCDC4: *Candida albicans CDC4*; CaMET3p: *C. albicans MET3* promoter; Dox: Doxycycline; CaCdc4: *C. albicans* Cdc4; ScCDC4: *S. cerevisiae CDC4*; ScCdc4: *S. cerevisiae* Cdc4; Met/Cys: Methionine and cysteine; YEPD: Yeast extract-peptone-dextrose; SD: Synthetic defined; CaURA3: *C. albicans URA3*; CaADH1: *C. albicans ADH1*.

## Competing interests

The authors declare that they have no competing interests.

## Authors’ contributions

CC, WCL, JCS, TLL conceived and designed the experiments. CC, WCL, and TLT performed the experiments. CC, WCL, JCS, and TLT analyzed the data. WCL, TLL, and TLT contributed reagents and materials. JCS wrote the paper. All authors read and approved the final manuscript.

## Supplementary Material

Additional file 1: Figure S1 N-terminal 85-amino acid of *Ca*Cdc4 is required for normal growth of *C. albicans*. Strains: BWP17, heterozygous null mutant *CaCDC4* +/-, M3*CaCDC4* +/- carrying *CaMET3*-full-length *CaCDC4*, and M3NT*CaCDC4* +/- carrying *CaMET3*-partial *CaCDC4* (capable of expressing N-terminal 85-amino acid of truncated *Ca*Cdc4). Cells of the strains were grown initially in SD medium without Met/Cys to saturation and were diluted to the same initial concentration. Cells were grown for 12 hrs in SD either with or without 2.5 mM Met/Cys (-Met/Cys or + Met/Cys) and at each 2-hr interval the cells were sampled to determine the optical density of 595 nm (O.D. 595) in which the growth curves could be plotted. Click here for file

Additional file 2: Figure S2 N-terminal 85-amino acid of *Ca*Cdc4 is required for suppression of yeast-to-filament transition in *C. albicans*. Cells of the strains were grown initially in SD medium without Met/Cys to saturation and were diluted to the same initial concentration. Cells were grown for 8 hrs in SD either with or without 2.5 mM Met/Cys (-Met/Cys or + Met/Cys). The images were visualized and recorded with a Nikon 50i microscope at 400× magnification. Bars represent 10 μm. The designations of strains are the same as in Additional file 1: Figure S1. Click here for file
