# Supplementary figures and images for: Dissection of the Candida albicans Cdc4 protein reveals the involvement of domains in morphogenesis and cell flocculation
Source: J Biomed Sci. 2013 Dec 20;20(1):97. doi: 10.1186/1423-0127-20-97 (PMC3878131; doi:10.1186/1423-0127-20-97)

## Slide 1
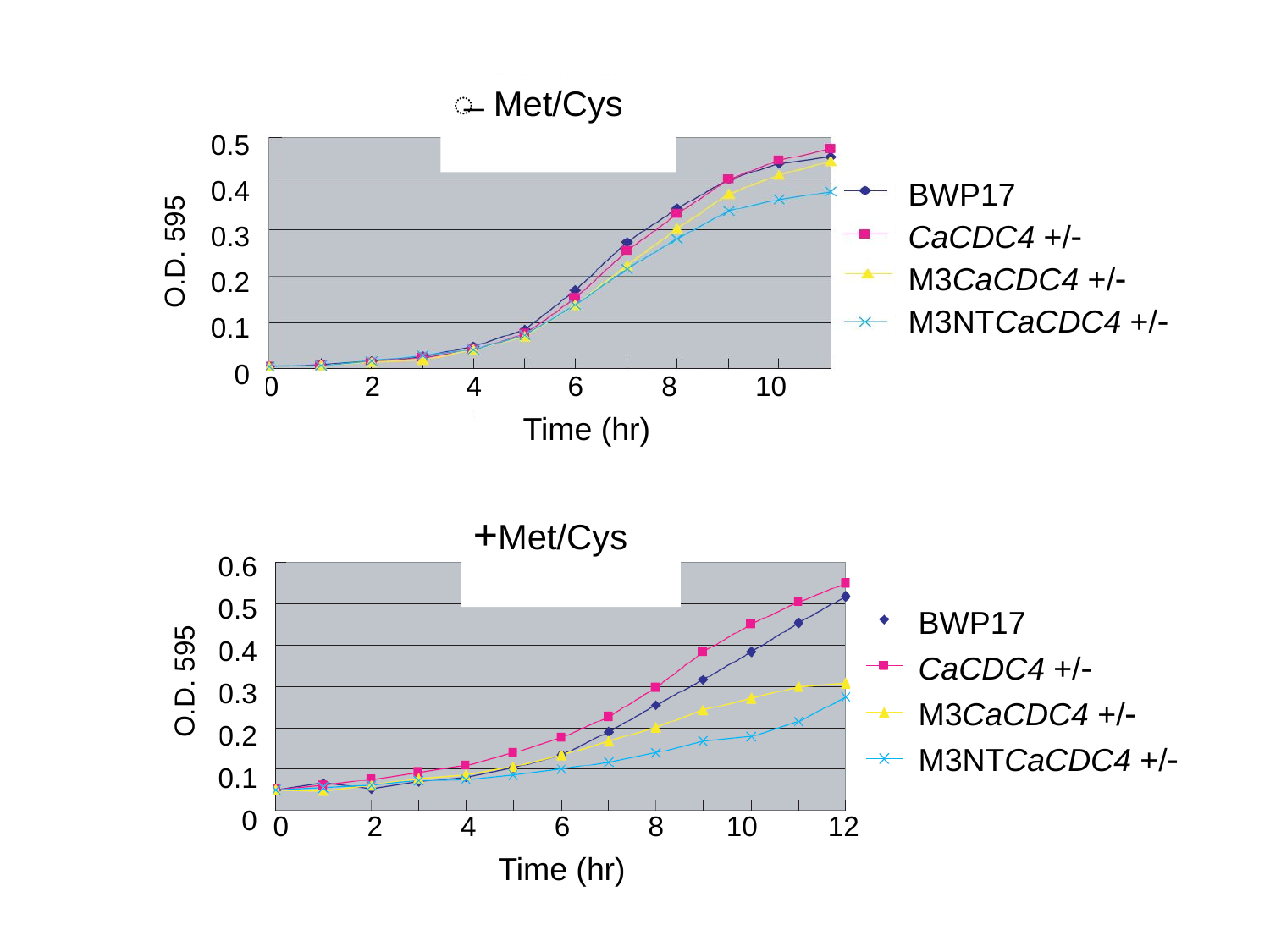

̶ Met/Cys
BWP17
CaCDC4 +/−
M3CaCDC4 +/−
M3NTCaCDC4 +/−
 Time (hr)
0.5
0.4
0.3
0.2
0.1
 0
O.D. 595
0 2 4 6 8 10
+Met/Cys
BWP17
CaCDC4 +/−
M3CaCDC4 +/−
M3NTCaCDC4 +/−
Time (hr)
0.60.5
0.4
0.3
0.2
0.1
 0
O.D. 595
0 2 4 6 8 10 12

Supplement: Additional file 1: Figure S1 — N-terminal 85-amino acid of CaCdc4 is required for normal growth of C. albicans. Strains: BWP17, heterozygous null mutant CaCDC4 +/-, M3CaCDC4 +/- carrying CaMET3-full-length CaCDC4, and M3NTCaCDC4 +/- carrying CaMET3-partial CaCDC4 (capable of expressing N-terminal 85-amino acid of truncated CaCdc4). Cells of the strains were grown initially in SD medium without Met/Cys to saturation and were diluted to the same initial concentration. Cells were grown for 12 hrs in SD either with or without 2.5 mM Met/Cys (-Met/Cys or + Met/Cys) and at each 2-hr interval the cells were sampled to determine the optical density of 595 nm (O.D. 595) in which the growth curves could be plotted. [file 1423-0127-20-97-S1.pptx]

## Slide 1
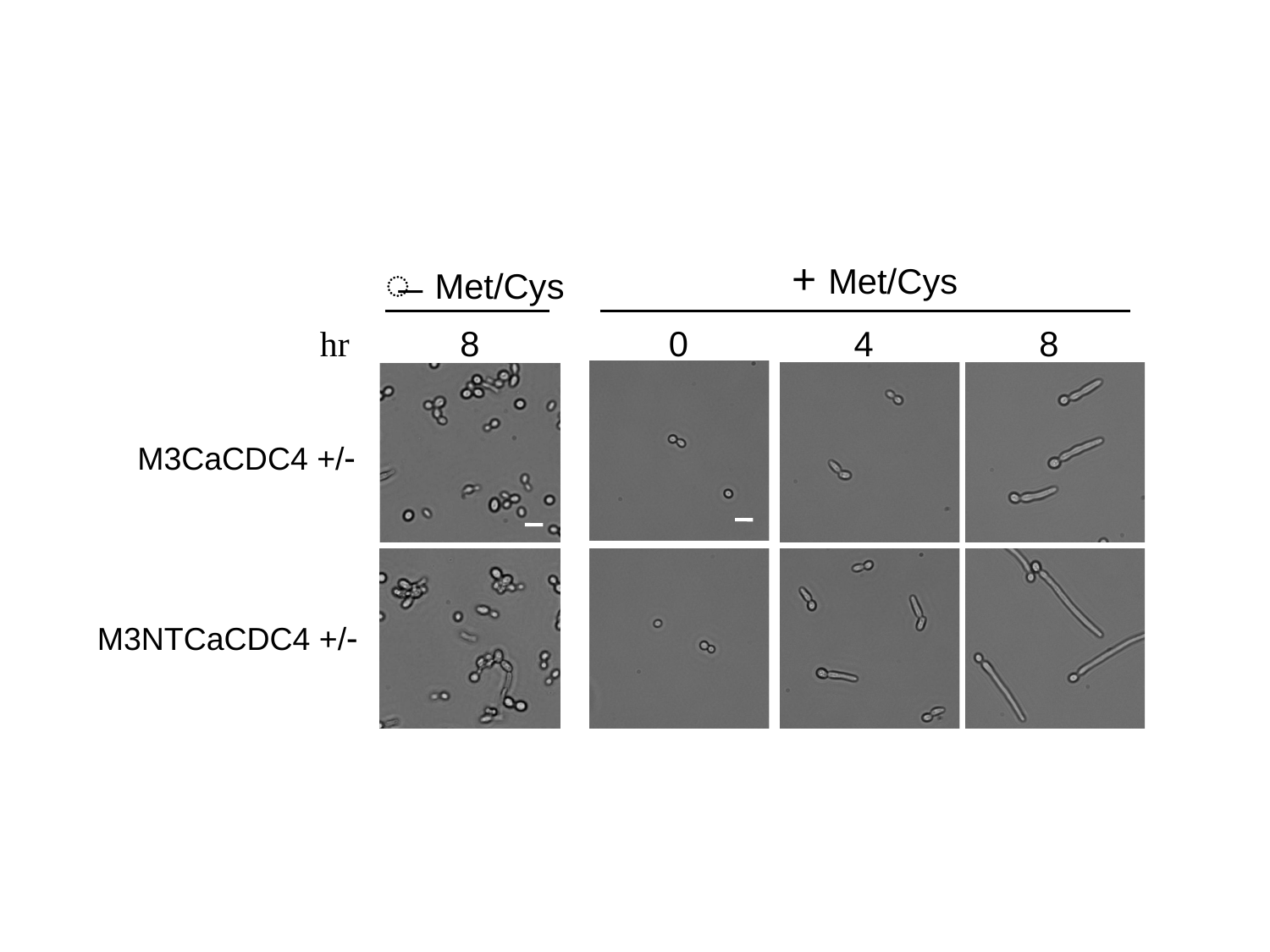

+ Met/Cys
̶ Met/Cys
8
0 4 8
hr
M3CaCDC4 +/−
M3NTCaCDC4 +/−

Supplement: Additional file 2: Figure S2 — N-terminal 85-amino acid of CaCdc4 is required for suppression of yeast-to-filament transition in C. albicans. Cells of the strains were grown initially in SD medium without Met/Cys to saturation and were diluted to the same initial concentration. Cells were grown for 8 hrs in SD either with or without 2.5 mM Met/Cys (-Met/Cys or + Met/Cys). The images were visualized and recorded with a Nikon 50i microscope at 400× magnification. Bars represent 10 μm. The designations of strains are the same as in Additional file 1: Figure S1. [file 1423-0127-20-97-S2.pptx]
